# Lattice SFRT in a geriatric patient with an initially inoperable leg bulky sarcoma: case report and literature review

**DOI:** 10.3389/fonc.2026.1932340

**Published:** 2026-09-03

**Authors:** Francesco Di Siervi, Bruno Curcio, Renato Cuocolo, Silvio Maffei, Carolina Mainardi, Immacolata A.M. Pilotti, Davide Di Gennaro

**Affiliations:** 1 Radiation Oncology Unit, University Hospital “San Giovanni di Dio e Ruggi d’Aragona”, Salerno, Italy; 2 Department of Medicine, Surgery and Dentistry “Scuola Medica Salernitana”, University of Salerno, Baronissi, Italy; 3 Medical Physics Unit, University Hospital “San Giovanni di Dio e Ruggi d’Aragona”, Salerno, Italy

**Keywords:** geriatric oncology, lattice radiation therapy (LRT), radiation oncology, soft tissue sarcoma, spatially fractionated radiation therapy (SFRT), pleomorphic liposarcoma

## Abstract

**Background:**

Management of inoperable bulky soft tissue sarcomas mostly in geriatric patients presents a significant clinical challenge due to high and hypoxia-induced radioresistance, proximity to dose-limiting Organs at Risk (OARs), and the need to minimize systemic toxicity. Spatially Fractionated Radiation Therapy (SFRT), specifically 3D Lattice Radiation Therapy (LRT), offers a novel approach to safely escalate dose within the tumor volume.

**Case presentation:**

85-year-old female presenting a massive (20.7 x 9.8 cm), inoperable high-grade pleomorphic liposarcoma (FNCLCC Grade 3) of the right thigh. A hybrid fractionation approach was employed, consisting of conventional Volumetric Modulated Arc Therapy (VMAT) (40 Gy in 20 fractions) to the Clinical Target Volume (CTV), concurrently integrated with a weekly Lattice boost of 10 Gy, for 3 times, targeting 4 specific series of spatial vertices (Sup1, Sup2, Inf1, Inf2) using a strip-field technique. Remarkably, physical examination during the fourth week revealed a dramatic reduction in tumor consistency and pain relief.

**Conclusion:**

The tailored hybrid treatment was exceptionally well tolerated with no severe acute skin toxicity. The 4-month follow-up MRI showed stable external dimensions with internal coagulative necrosis, allowing adjuvant surgery. This case highlights the feasibility, safety, and tolerability of a response-adapted weekly hybrid LRT approach for bulky extremity sarcomas especially in frail populations. We also provide a brief review of the emerging literature on SFRT/LRT in bulky sarcomas and frail patients.

## 1 Introduction

Bulky extremity sarcomas (> 10 cm) typically exhibit central necrosis and profound hypoxic cores, rendering them highly resistant to conventionally fractionated radiotherapy (1, 2). Furthermore, delivering a homogeneous radical dose to large volumes invariably exceeds the tolerance constraints of adjacent OARs and healthy peripheral tissues, a risk exponentially higher in geriatric patients. Lattice Radiation Therapy (LRT), an advanced 3D implementation of Spatially Fractionated Radiation Therapy (SFRT), may overcome this dosimetric paradox increasing therapeutic ratio (3–5). By generating high-dose spatial “vertices” interspaced with low-dose “valleys, “ LRT is hypothesized to capitalize on indirect radiobiological mechanisms - such as endothelial apoptosis, the bystander effect, and immune system priming, while strictly adhering to normal tissue constraints (3, 6, 7). Usually, LRT is delivered as a single large fraction prior to standard RT, while we preferred a different and more adaptive hybrid approach in a geriatric patient: a weekly LRT boost was integrated into a conventional fractionation schedule (8).

## 2 Case presentation

### 2.1 Patient information and clinical findings

An 85-year-old female (height 150 cm, weight 50 kg, ECOG Performance Status 4, chair-bound) presented with a painful mass in the anterior compartment of the right thigh. At presentation, the patient was completely non-ambulatory, permanently wheelchair-bound, and suffering from excruciating, intractable movement-induced pain. Baseline MRI revealed a bulky mass measuring 20.7 x 9.8 cm. Core needle biopsy confirmed a high-grade pleomorphic liposarcoma (FNCLCC Grade 3, Ki-67 proliferation index ~60%) (9). Staging CT confirmed T4 N0 M0 (Stage III, AJCC 8th Edition) disease without distant metastases. Major comorbidities included diabetes mellitus, senile dementia, history of deep vein thrombosis (DVT) in the contralateral lower extremity, right hip endoprosthesis and prior right carotid thromboendarterectomy. Due to the massive tumor and the patient’s advanced age, surgical resection was at the time deemed contraindicated (10).

### 2.2 Treatment planning and dosimetry

**Figure 1**.

**Figure 1:**
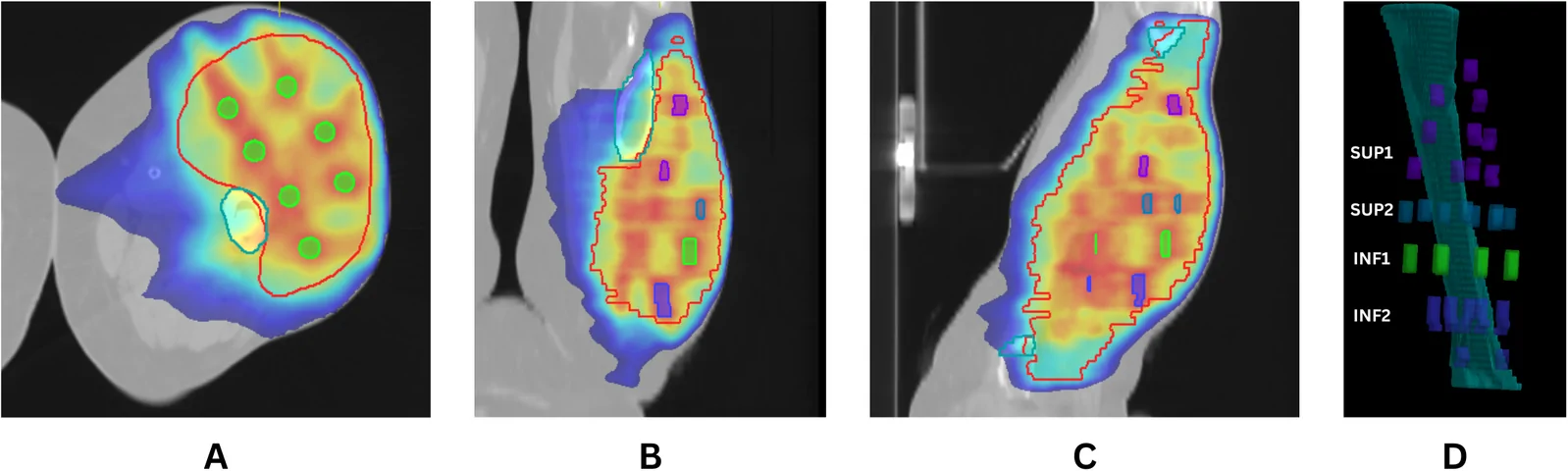
Spatially fractionated/lattice radiation therapy (LRT) planning geometry and dose distribution. 3D planning rendering from Monaco TPS illustrating the strip-field technique and fusiform grid architecture utilized for the hybrid treatment plan: **(A–C)** Axial, coronal, and sagittal CT views showing high-dose lattice vertices precisely positioned within the Clinical Target Volume (CTV) and the corresponding cumulative dose distribution, demonstrating effective dose sparing of adjacent healthy tissues and the right femur. **(D)** 3D spatial reconstruction illustrating the vertical stack configuration of the four high-dose lattice vertex groups (Sup1, Sup2, Inf1, Inf2) arranged in a fusiform grid relative to the contoured right femur (cyan structure).

Local radiation oncology tumor board recommended a hybrid LRT approach. The treatment plan was generated using the Monaco Treatment Planning System (Elekta) utilizing a Monte Carlo calculation algorithm to ensure accuracy in high-gradient regions.

#### 2.2.1 Target and lattice geometry

Gross Tumor Volume (GTV) was defined as the palpable and radiologically visible mass on MRI. The Clinical Target Volume (CTV) measured 1417.6 cm³ and was defined by expanding the GTV by 4 cm craniocaudally and axially from the subcutaneous tissue up to ~2 cm beyond the tumor margin, manually adjusted to spare the femoral nerve bundle and skin. A 5 mm uniform margin was added to create the PTV. The right femur was contoured as an organ at risk along the segment adjacent to the target, extending cranio-caudally by 2 cm beyond the PTV limits. Four vertex groups (Sup1, Sup2, Inf1, Inf2) arranged in a 3D fusiform grid architecture were generated within the central core of the GTV. Each vertex was standardized to an equivalent diameter of 1.0 cm with a center-to-center spacing ranging from 2.5 to 3.0 cm (5, 11). The cumulative volume of the Lattice vertices was 43.1 cm³ (four groups ranging from 9.3 to 12.1 cm³), strictly encompassing 3.0% of the total CTV.

#### 2.2.2 Dosimetry

The treatment was designed as a response-adapted protocol: the initial prescription consisted of a baseline dose of 50 Gy in 25 fractions to the CTV, with a pre-planned clinical and dosimetric evaluation at week 4 (after 40 Gy in 20 fractions) to assess tumor response and potential early cessation, aiming to minimize late-onset toxicity in this frail patient. Concurrently, the CTV received an integrated weekly boost of 10 Gy (8 Gy from Lattice vertices + 2 Gy from the baseline VMAT plan) for a total of three fractions. Dosimetric characterization of the Lattice component alone yielded a mean vertex peak dose of 25.94 Gy and an inter-vertex valley dose of 13.72 Gy, corresponding to a Peak-to-Valley Dose Ratio (PVDR) of 1.89 and a Volume Dose Ratio (VDR = D10%/D90%) to the GTV of 1.403 (12). Radiobiological calculation for the soft tissue sarcoma target, the 24 Gy Lattice boost yielded an EQD2 (α/β = 5 Gy) of 44.57 Gy and a BED (α/β = 5 Gy) of 62.40 Gy (3). For late-responding OARs, the boost yielded an EQD2 (α/β = 3 Gy) = 52.80 Gy and a BED (α/β = 3 Gy) = 88.00 Gy. Cumulative plan evaluation confirmed excellent normal tissue sparing, maintaining the mean dose to the contoured right femur (209.6 cm^3^, contoured 2 cm superiorly and inferiorly beyond the PTV) at 38.48 Gy (Max dose 64.04 Gy), with a *V*_40Gy_ of 49.64% (104.04 cm^3^) (13).

### 2.3 Therapeutic intervention

**Figure 2**.

**Figure 2:**
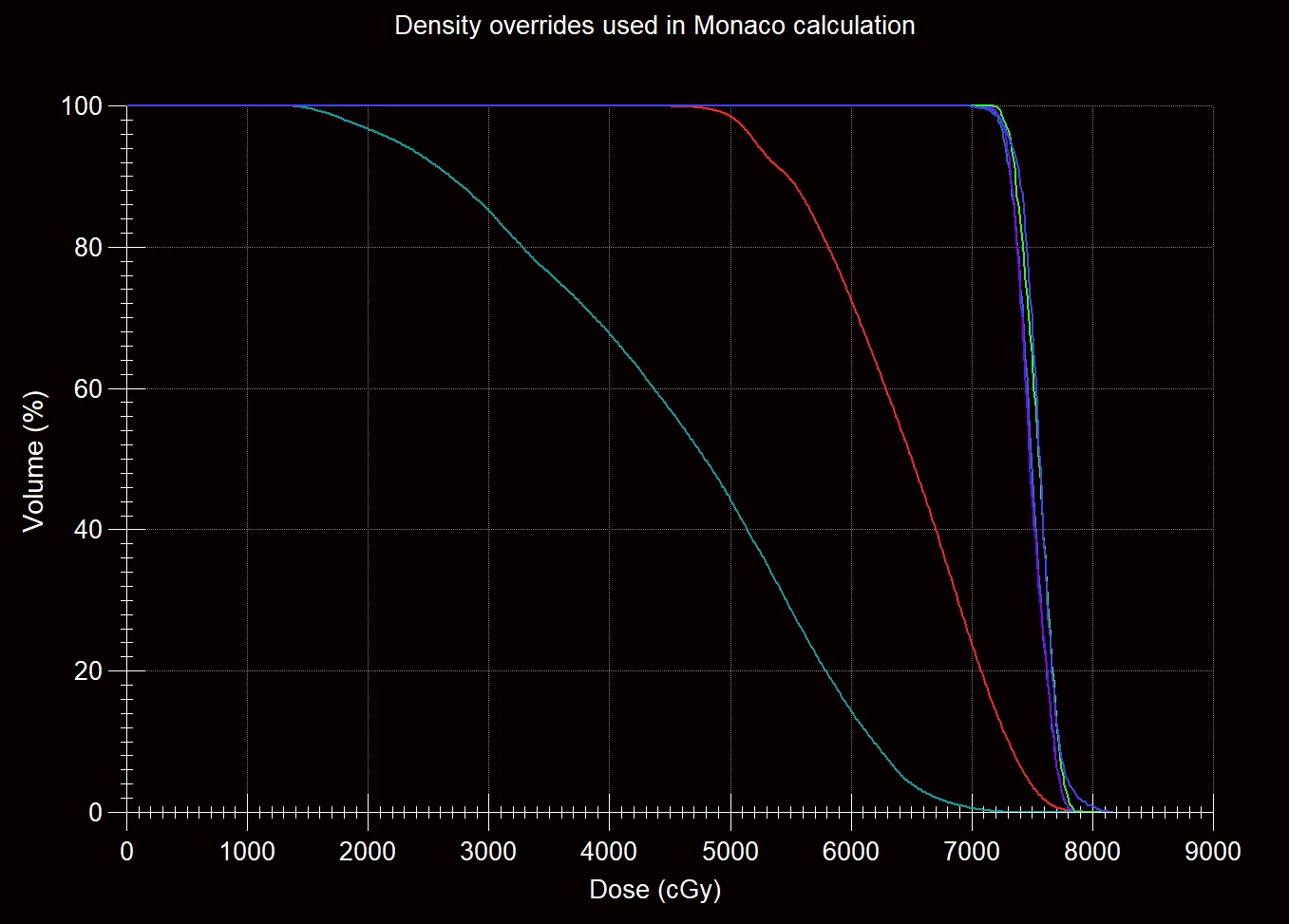
Dose-volume histogram (DVH) analysis of the hybrid LRT treatment. • Cyan curve: OAR (Femur) demonstrating effective dose sparing (mean dose 38.48 Gy), maintaining values well within tolerance thresholds. • Red curve: CTV (Clinical Target Volume) receiving the prescribed baseline dose coverage. • Right-sided curves: High-dose Lattice vertices (Sup1, Sup2, Inf1, Inf2), receiving a cumulative mean peak dose of 64.86 Gy. Due to virtually identical dose distributions across all four vertex groups, individual DVH curves exhibit graphic overlap on the right side of the plot.

Treatment was delivered using a Versa HD linear accelerator (Elekta) via VMAT. Image-guided radiotherapy (IGRT) with daily Cone Beam CT (CBCT) was strictly utilized, aided by pelvic tattoos and a Combileg immobilization system, to ensure sub-millimetric setup accuracy.

Remarkably, starting from the second week of RT, the patient experienced significant pain reduction. By the end of the third week, accompanied by marked tumor softening and relief of mechanical pressure, the patient regained independent/guided ambulation, improving her functional status from baseline ECOG 4 to an ambulatory ECOG 2. This dramatic early analgesic and functional recovery facilitated daily setup, sub-millimetric CBCT alignment, and overall treatment tolerance.

Given this marked clinical softening, and in accordance with the pre-planned response-adapted strategy, the multidisciplinary tumor board decided to halt the baseline radiotherapy course early at 40 Gy in 20 fractions as a pragmatic toxicity-avoidance measure in this severely frail patient (ECOG 4). All scheduled high-dose Lattice vertex boosts (10 Gy weekly for 3 fractions) were successfully delivered as planned.

Post-treatment cumulative dosimetric evaluation confirmed a total D95% to the CTV of 38.8 Gy. The high-dose Lattice vertices achieved a mean dose of 64.86 Gy (Max dose 70.82 Gy). Considering an α/β ratio of 5 Gy for soft tissue sarcoma, the 24 Gy Lattice boost alone provided an EQD2 of 44.57 Gy and a BED of 62.40 Gy, achieved without exceeding tolerance thresholds of surrounding normal tissues (14).

## 3 Follow-up and outcomes

Despite the massive target volume and the patient’s frailty, the hybrid treatment was exceptionally well tolerated. The patient experienced no high-grade (CTCAE ≥ Grade 2) acute skin toxicity or significant functional impairment (15).

**Figure 3**.

**Figure 3:**
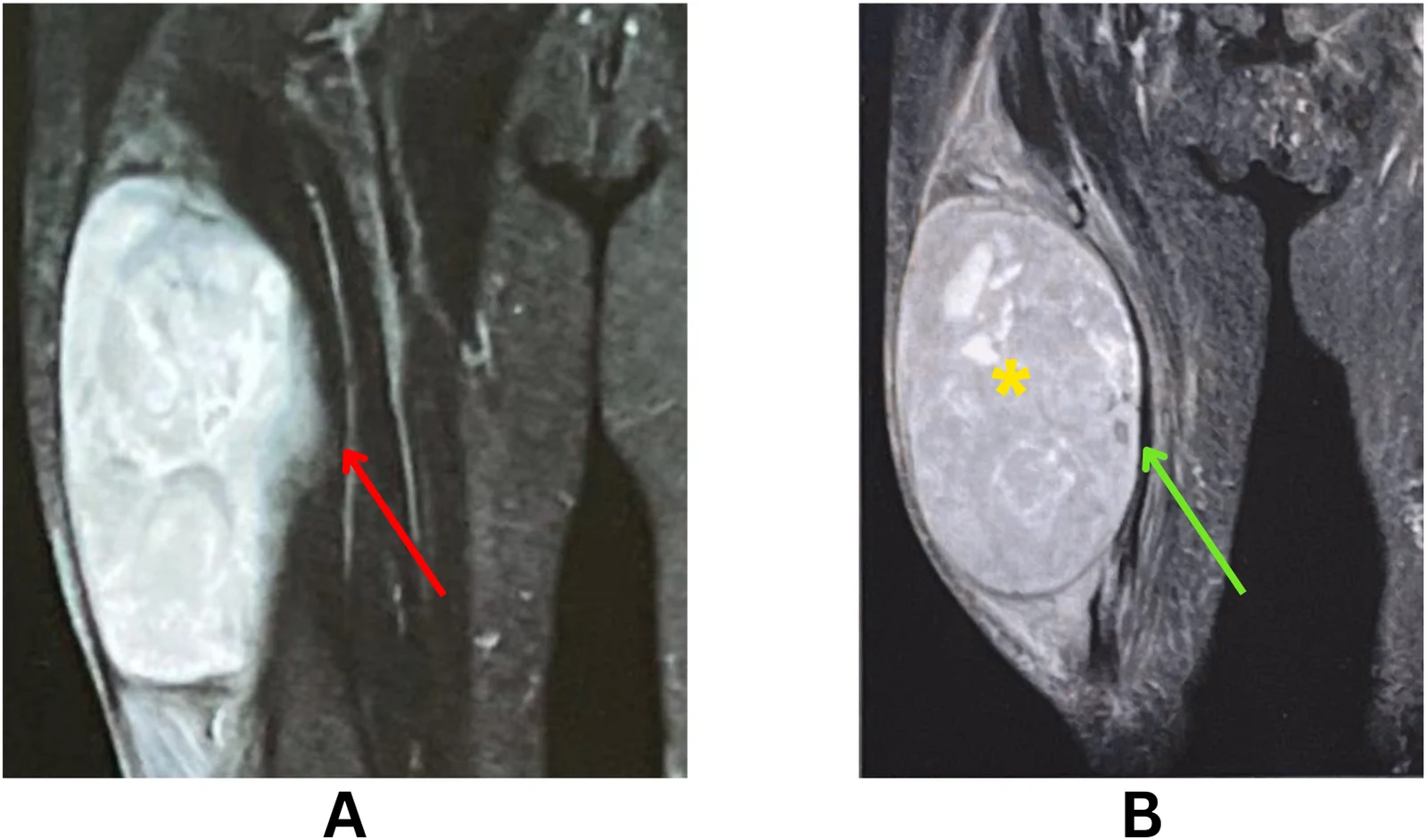
Comparative pre- and post-radiotherapy MRI (coronal T2-STIR sequences). **(A)** Baseline MRI: Massive soft-tissue sarcoma showing marked T2-STIR dishomogeneity with direct anatomical adherence and proximity along the femoral axis (red arrow). **(B)** Spatially matched follow-up MRI: 4-month post-RT evaluation demonstrating extensive intratumoral T2-hyperintensity corresponding to 95% pathologically confirmed liquefactive/coagulative necrosis (yellow asterisk). Note the appearance of a distinct low-signal fibrous demarcation plane separating the lesion from the femoral periosteum (green arrow), facilitating subsequent surgical resection. Minor visual proportion variations reflect acquisition Field-of-View (FOV) settings and treatment-induced intratumoral liquefaction/edema, while physical external tumor dimensions remained stable.

At the 4-month follow-up MRI, the mass measured 16.96 x 9.65 cm. While the external dimensions remained quite stable, a clinically acceptable endpoint in giant sarcomas, the MRI revealed profound internal structural remodelling. A significant alteration in T2/STIR signal intensity was observed, consistent with extensive therapy-induced coagulative necrosis (16).

Following this favorable anatomical response and clearance from the femoral periosteum, the patient underwent successful surgical resection six months after completion of radiotherapy. Histopathological examination of the resected specimen confirmed complete resection with negative surgical margins (R0 resection) and demonstrated 95% quantitative intratumoral coagulative necrosis with only 5% residual viable tumor cellularity surrounded by reactive fibrotic tissue (17).

The postoperative course was uneventful, with no major wound healing complications or adverse events. At the last available follow-up, the patient remained free of clinical evidence of local recurrence or distant progression. Unfortunately, 18 months post-surgery, the patient suffered an accidental traumatic fall at home resulting in a mid-diaphyseal right femur fracture. Notably, the patient was already a carrier of a right hip endoprosthesis due to a femoral neck fracture sustained more than 2 years prior to RT. The new fracture occurred approximately 8 cm distal to the pre-existing prosthetic stem in a mid-diaphyseal segment receiving a local dose of approximately 45 Gy, roughly 1.0 cm away from the high-dose Lattice vertices. Radiographs confirmed well-calcified cortical bone compatible with age, indicating a trauma-induced origin rather than a classical radiation-associated insufficiency fracture.

**Figure 4**.

**Figure 4:**
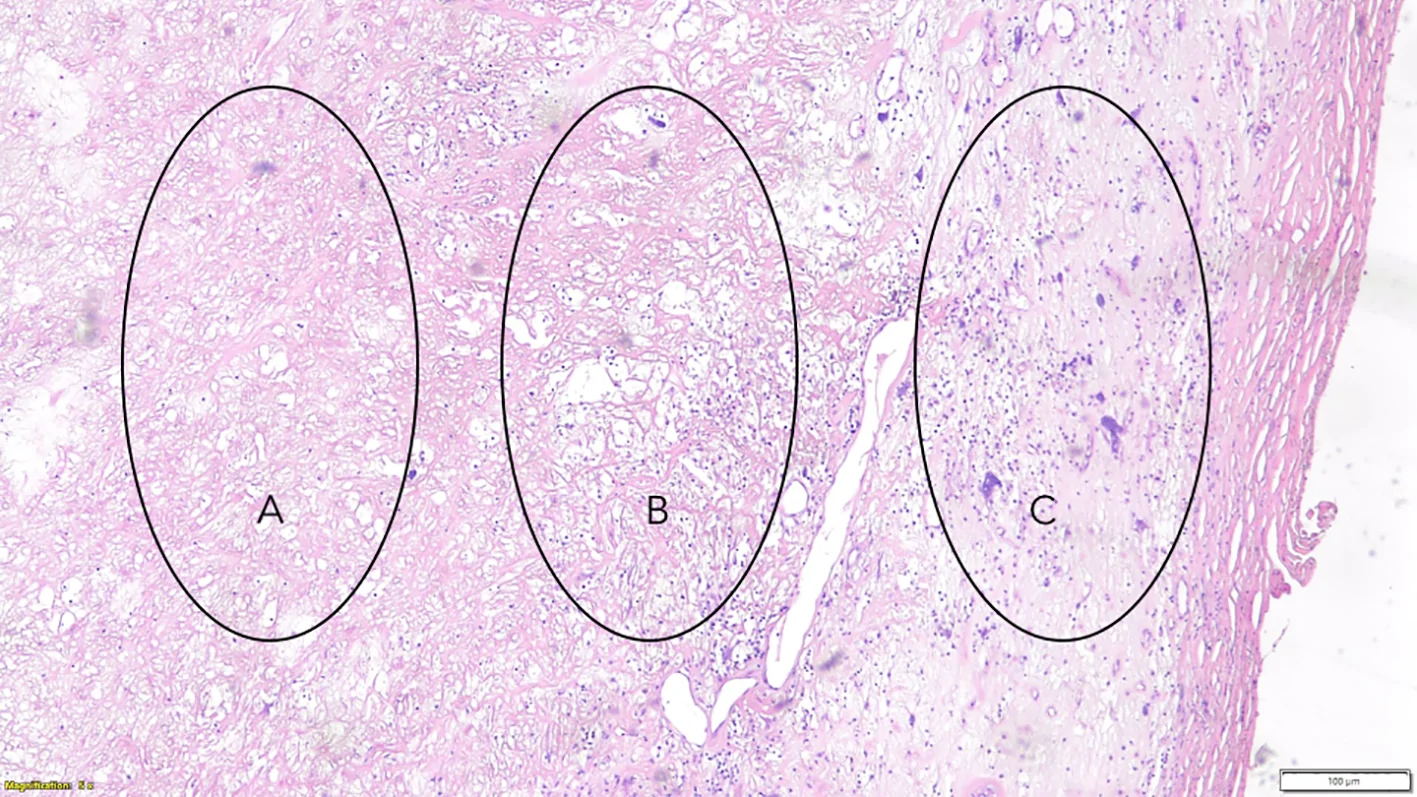
Histopathologic response after hybrid lattice-VMAT treatment. Hematoxylin and Eosin stain, 5x magnification. Extensive (95%) central coagulative necrosis **(A)**, transition zones between necrotic and residual mesenchymal tissue **(B)**, and peripheral fibrotic reparative stromal changes (<5% viable neoplastic tissue) and negative surgical margins (R0) **(C)** were observed in the resected specimen.

## 4 Discussion

### 4.1 Radiobiological rationale of SFRT/LRT

Bulky soft tissue sarcomas often harbor hypoxic subvolumes (with expression of HIF-1α) and pre-existing central necrosis, features that contribute to marked radioresistance when a homogeneous dose is delivered with conventional fractionation (1, 2, 18). In addition, the delivery of a radical uniform dose to very large target volumes is frequently limited by the tolerance of surrounding normal tissues, particularly in frail or elderly patients with reduced physiological reserve (10). SFRT, and in particular LRT, was developed to address both of these constraints by creating a geometric pattern of high-dose vertices surrounded by lower-dose valleys within the tumor volume (3–5).

This spatial modulation is thought to act through mechanisms that extend beyond direct tumor cell kill alone. High-dose vertices may induce localized endothelial and vascular damage, promote coagulative necrosis, and trigger bystander and abscopal-like signaling within the tumor microenvironment (6, 7). At the same time, the preservation of lower-dose regions between vertices may help maintain acceptable normal tissue tolerance while still generating a biologically meaningful intratumoral cascade. This model is particularly attractive in large, hypoxic, and heterogeneous tumors, where conventional dose escalation is often not feasible.

In the present case, this rationale guided a pragmatic treatment strategy considering the patient’s advanced age (85 years), extreme tumor bulk (20.7 x 9.8 cm), severe frailty (ECOG 4), and intimate proximity of the femur. Under these circumstances, a response-adapted hybrid approach combining conventionally fractionated VMAT with integrated weekly Lattice boosts allowed selective dose intensification to the central tumor volume while minimizing high-dose exposure to surrounding critical normal tissues. This regimen aimed to achieve safe palliative local control and symptom relief without inducing severe acute toxicity in a high-risk geriatric patient.

### 4.2 Evidence on SFRT/LRT for bulky sarcomas: brief literature review

The available literature on SFRT/LRT suggests that this technique may be particularly useful in bulky and radioresistant neoplasms, where standard uniform irradiation is limited by both tumor biology and normal tissue constraints (3–5, 12). Most published experiences describe encouraging clinical responses, symptomatic improvement, and acceptable toxicity profiles, especially when LRT is used as a boost integrated with external beam radiotherapy or as a strategically intensified component of treatment in large tumor masses (3, 4, 8, 11, 18, 19). Although the evidence remains largely based on retrospective series, technical reports, and selected case experiences, the consistency of these observations supports the growing interest in this approach.

Specific evidence in soft tissue sarcomas is still limited, but the rationale appears especially strong in this setting (8, 14, 19). Sarcomas frequently present as large, heterogeneous lesions with necrotic and hypoxic cores, and radiological or pathological evidence of internal treatment response may be more informative than external dimensional shrinkage alone. In this regard, reports on preoperative radiotherapy for soft tissue sarcoma have shown that substantial internal remodeling, coagulative necrosis, and histopathological response can occur even when gross tumor dimensions remain relatively stable on imaging (16, 17). This concept is highly relevant to the interpretation of response after SFRT/LRT in giant sarcomas.

Another important gap in the literature concerns frail and geriatric patients, who are often underrepresented in prospective radiotherapy studies despite being among those most likely to benefit from individualized, toxicity-sparing strategies (10). In this context, the present case adds clinical observations by showing that a weekly hybrid lattice approach can be delivered safely in an 85-year-old patient with a very large initially inoperable extremity sarcoma, achieving rapid symptom relief, marked internal tumor necrosis, and eventual conversion to surgical resectability, in line with emerging data on SFRT/LRT in bulky soft-tissue sarcomas (8, 12, 18, 19). Therefore, beyond confirming the feasibility of SFRT/LRT in bulky disease, this case supports its potential role as a personalized option in highly selected elderly patients for whom standard aggressive approaches may be poorly tolerated or initially deemed impractical.

### 4.3 Implications of our case for clinical practice

This case suggests that a weekly hybrid LRT approach may be a feasible and well-tolerated option in selected frail patients with bulky extremity sarcoma (4). Delivering a radical homogeneous dose to a 1417.6 cm3 volume in an 85-year-old patient would traditionally be expected to substantially increase the risk of systemic and local toxicity, further complicated by the proximity of the adjacent femur. However, the Monte Carlo-optimized spatial geometric arrangement of the lattice vertices allowed the healthy peripheral tissues to remain in low-dose valleys (5, 8).

The rapid clinical softening of the mass, followed by the extensive central necrosis observed on follow-up MRI and confirmed on post-surgical histology (95% necrosis), is consistent with treatment-induced tumor degradation. The presence of marked STIR hyperintensity post-treatment reflects profound structural remodeling within the lesion (16, 17).

Crucially, the clinical decision to halt the baseline radiotherapy course early at 40 Gy in 20 fractions was a pragmatic, multidisciplinary choice driven by the patient’s severe frailty (ECOG 4) and rapid clinical response, serving as an effective toxicity-avoidance strategy rather than a formal de-escalation protocol. This response-adapted model provided a tolerable and personalized palliative option that ultimately enabled a safe surgical resection.

### 4.4 Study limitations

This report has limitations that must be acknowledged. First, as a single-case experience with an observational design, the findings cannot be generalized to broader clinical practice. Second, because the weekly Lattice boosts were delivered concurrently with a conventional 40 Gy VMAT regimen, the specific therapeutic contribution of spatial fractionation cannot be isolated or quantitatively separated from the radiation response induced by standard VMAT. Finally, proposed radiobiological mechanisms such as immune activation, bystander effects, or vascular collapse remain theoretical hypotheses derived from general literature and cannot be directly confirmed by our clinical data.

## 5 Patient perspective

The patient reported meaningful pain relief and improved physical comfort during therapy, particularly following the initial weeks of treatment. She tolerated the combined hybrid regimen remarkably well without acute high-grade toxicities, ultimately achieving significant clinical benefit that allowed surgical evaluation.

## 6 Conclusion

**Figure 5**.

**Figure 5:**
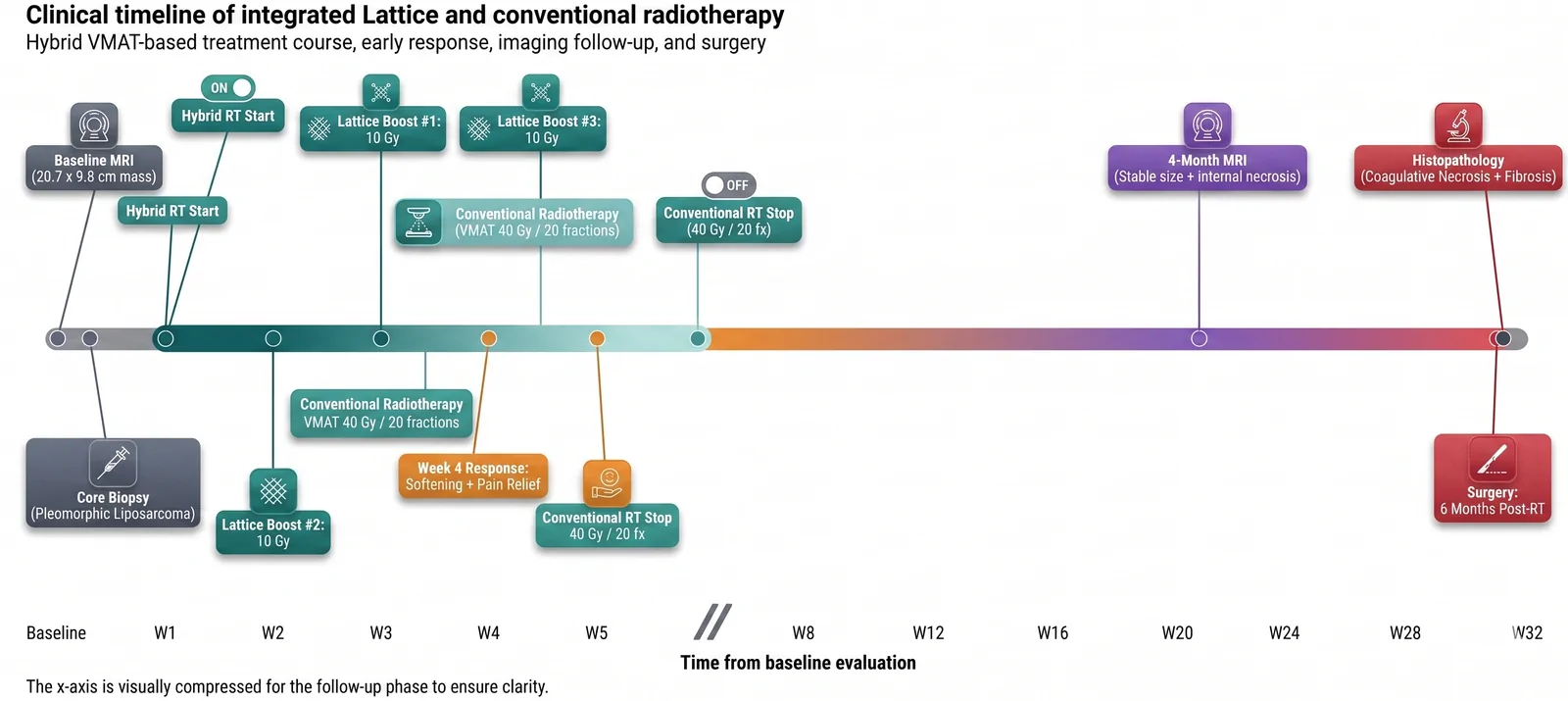
Clinical timeline of the hybrid treatment course. Conventional radiotherapy was delivered as VMAT to 40 Gy in 20 fractions, with three integrated weekly Lattice boosts of 10 Gy during the baseline course. Palpable tumor softening and pain relief were observed during week 4, prompting protective early cessation. Follow-up MRI at 4 months confirmed marked internal necrosis, and surgery at 6 months achieved R0 resection.

Integrated weekly Lattice Radiation Therapy represents a feasible, well-tolerated, and useful approach for the conservative management of initially inoperable bulky soft tissue sarcomas in frail geriatric patients (8). It offers a pragmatic solution to the dosimetric paradox of delivering high target doses to large hypoxic volumes while strictly respecting normal tissue tolerance constraints (3, 9, 10). The response-adapted decision to halt the baseline regimen early at 40 Gy served as an effective toxicity-avoidance strategy, achieving substantial internal tumor response and symptom control without acute severe side effects, ultimately enabling successful surgical resection.

## Data Availability

The raw data supporting the conclusions of this article will be made available by the authors, without undue reservation.
